# Supplementary material for: Genome-wide CRISPR knockout cell screening platform for the disease vector tick species Ixodes scapularis
Source: bioRxiv. 2026 May 7:2026.05.05.721418. Preprint. [Version 1] doi: 10.64898/2026.05.05.721418 (PMC13174539; doi:10.64898/2026.05.05.721418)
Supplement: 1 [file NIHPP2026.05.05.721418V1-supplement-1.pdf]

bioRxiv preprint doi: https://doi.org/10.64898/2026.05.05.721418; this version posted May 7, 2026. The copyright holder for this preprint (which was not certified by peer review) is the author/funder, who has granted bioRxiv a license to display the preprint in perpetuity. It is made available under aCC-BY 4.0 International license.

Supplemental Figure Legends

Supplemental Figure 1: Molecular tool analyses in multiple *I. scapularis* cell lines. (A) – (E) Comparison of pol II promoters in five *I. scapularis* cell lines. Graphs of expression of firefly luciferase from the indicated pol II promoter normalized to CAG-Renilla luciferase. *D. mel* actin, *Drosophila melanogaster* Actin5c promoter. (F) Assay of pol III activity in RMCE+ Cas9+ ISE6 DsRed cells. Graph of DsRed levels following expression of a DsRed-targeting sgRNA with the 025 *I. scapularis* pol III promoter (only 025 was tested). (G) Evidence of recombination mediated cassette exchange (RMCE) in RMCE+ Cas9+ ISE6 DsRed cells. An attB-containing CAG-GFP cassette (pLib025-GFP) was transfected into RMCE+ Cas9+ ISE6 DsRed cells in the presence (left) or absence (right) of a plasmid expressing PhiC31 integrase. Shown are representative flow cytometry plots. GFP-positive cells are observed in the population with PhiC31 (left), indicating successful cassette exchange.

Supplemental Figure 2: Comparison of expression patterns in *I. scapularis* cultured cell lines. Heatmap of the Pearson correlation for gene expression in IDE2, ISE6, ISE18, and a pool of RMCE+ ISE18 DsRed cells enriched for. Four (IDE2, ISE18) or three (ISE6, RMCE+ ISE18 DsRed) replicate samples are shown (S1, S2, etc.). Highest correlations are seen within replicate samples from the same cell line, and between ISE18 and RMCE+ ISE18 DsRed, and ISE6 and ISE18 are more similar to one another than either is to IDE2.

Supplemental Figure 3: Summary of *I. scapularis* ISE18 CRISPR-Cas9 knockout (KO) cell screen workflows. (A) Single guide RNAs (sgRNAs) were designed using CRISPR GuideXpress, synthesized, and cloned into an *I. scapularis* pol III-driven sgRNA expression vector. (B) Cells were transfected with the sgRNA library and enriched for sgRNA integration via FACS. Samples of outgrown KO cell libraries were collected for the ‘drop-out’ (fitness) gene screen. (C) For treatment resistance screens (selection assays), cells from the FACS-enriched outgrown KO cell library were cultured in the absence (control) or presence of a cytotoxic treatment. The specific treatment regimen (e.g., timing of treatment, continuous treatment or

bioRxiv preprint doi: https://doi.org/10.64898/2026.05.05.721418; this version posted May 7, 2026. The copyright holder for this preprint (which was not certified by peer review) is the author/funder, who has granted bioRxiv a license to display the preprint in perpetuity. It is made available under aCC-BY 4.0 International license.

cells were allowed to recover in standard media between treatments, etc.) differed for different screens. Following the treatment regimen, cells were collected for NGS.

Supplemental Figure 4: AlphaFold modeling of *I. scapularis* Ctr1A. (A) Evaluation of *I. scapularis* Ctr1A oligomeric states predicted by AlphaFold3. The panels display metrics averaged across five independent structural models for each tested subunit stoichiometry. Visualizations include the Predicted Aligned Error (PAE) map, Local Interaction Area (LIA) and contact-filtered LIA (cLIA) maps, and heatmaps for the Local Interaction Score (LIS), integrated LIS (iLIS), and interface predicted Template Modeling (ipTM) score. Low PAE (blue color) is suggestive of confident interactions. The trimeric assembly exhibits the highest iLIS and ipTM values, suggesting that it is the most structurally stable and confident oligomerization state. Plots generated as described in **Methods**. (B) AlphaFold3 predictions of Ctr1A interactions with different ions. The scatter plot compares the average iLIS against the ipTM score. Higher iLIS and ipTM values indicate a stronger predicted interaction and higher confidence in the protein-ion interface geometry, respectively. For each ion condition, five independent models were generated and plotted. The prediction with Cu^{2+} exhibits the strongest interaction among the tested ions. (C-D) AlphaFold3-predicted structure of the Ctr1a trimer alone (C) or in complex with Cu^{2+} (D). The structure is colored by pLDDT, which ranges from 0 to 100, where higher values (blue) indicate greater confidence in the local structure and lower values (orange) indicate lower confidence. The addition of Cu^{2+} increases the predicted rigidity of the trimer, as indicated by higher overall pLDDT scores. The red arrow points to the copper ion. The N-terminal region (residues 1–90) had a high PAE and was omitted for clarity.

Supplemental Figure 5: AlphaFold modeling of *I. scapularis* Cup2 and Cup2-Ctr1A heterotrimeric complexes. (A) Top, Cup2 homotrimer structure colored by pLDDT. Orange and yellow pLDDT indicate low-confidence predictions. Bottom, PAE map of the Cup2 homotrimer prediction. The low pLDDT and high PAE observed for the Cup2 homotrimer prediction suggests it is unlikely this structure forms. (B) Top, 1 x Ctr1A and 2 x Cup2 heterotrimer structure prediction colored by pLDDT. Compared to the Cup2 homotrimer, this heterotrimer has a higher pLDDT (sky blue), suggesting possible heterotrimer complex

bioRxiv preprint doi: https://doi.org/10.64898/2026.05.05.721418; this version posted May 7, 2026. The copyright holder for this preprint (which was not certified by peer review) is the author/funder, who has granted bioRxiv a license to display the preprint in perpetuity. It is made available under aCC-BY 4.0 International license.

formation. Center, the same structure with a copper ion (red arrow). Note the darker blue (high-confidence in pLDDT, stable structure). Right, the same structure with a copper ion (red arrow), top view. Bottom, corresponding PAE maps for the heterotrimer without (left) or with (right) a copper ion. The heterotrimer with a copper ion shows lower PAE (darker blue), suggesting more stable complex (C) Top, 2 x Ctr1A and 1 x Cup2 heterotrimer structure prediction colored by pLDDT. Compared to the Cup2 homotrimer, this heterotrimer has a higher pLDDT (sky blue), suggesting a relatively stable structure. Center, the same structure with a copper ion (red arrow). Note the darker blue (high-confidence in pLDDT, stable structure). Right, the same structure with a copper ion (red arrow), top view. Bottom, corresponding PAE maps for the heterotrimer without (left) or with (right) a copper ion. The heterotrimer with a copper ion shows lower PAE (darker blue), suggesting a more stable complex. (E, F), Predicted ion preference for Ctr1A-Cup2 heterotrimers.

Supplemental Figure 6: Genome-wide screen for resistance to Antimycin A. Graph comparing data from the two replicate screens. *I. scapularis ND-B17.2* (NCBI Gene ID LOC8025722) is a predicted ortholog of human *NDUFA12*; *Ppat-Dpck* (LOC8025601), predicted ortholog of *COASY*; LOC8041162, predicted ortholog of *AKIR1*.

Supplemental Figure 7: Quantitative real-time PCR (RT-qPCR) analysis of RNAi knockdown of *PIG-A* in ISE6 cells. Relative *PIG-A* mRNA expression was measured in ISE6 cells transfected with siRNAs targeting a non-targeting control siRNA (scPIG-A) or targeting the *PIG-A* gene (siPIG-A). Transcript levels were quantified by RT-qPCR and normalized using Actin as a reference gene.

bioRxiv preprint doi: https://doi.org/10.64898/2026.05.05.721418; this version posted May 7, 2026. The copyright holder for this preprint (which was not certified by peer review) is the author/funder, who has granted bioRxiv a license to display the preprint in perpetuity. It is made available under aCC-BY 4.0 International license.

Supplemental Figures

Supplemental Figure 1

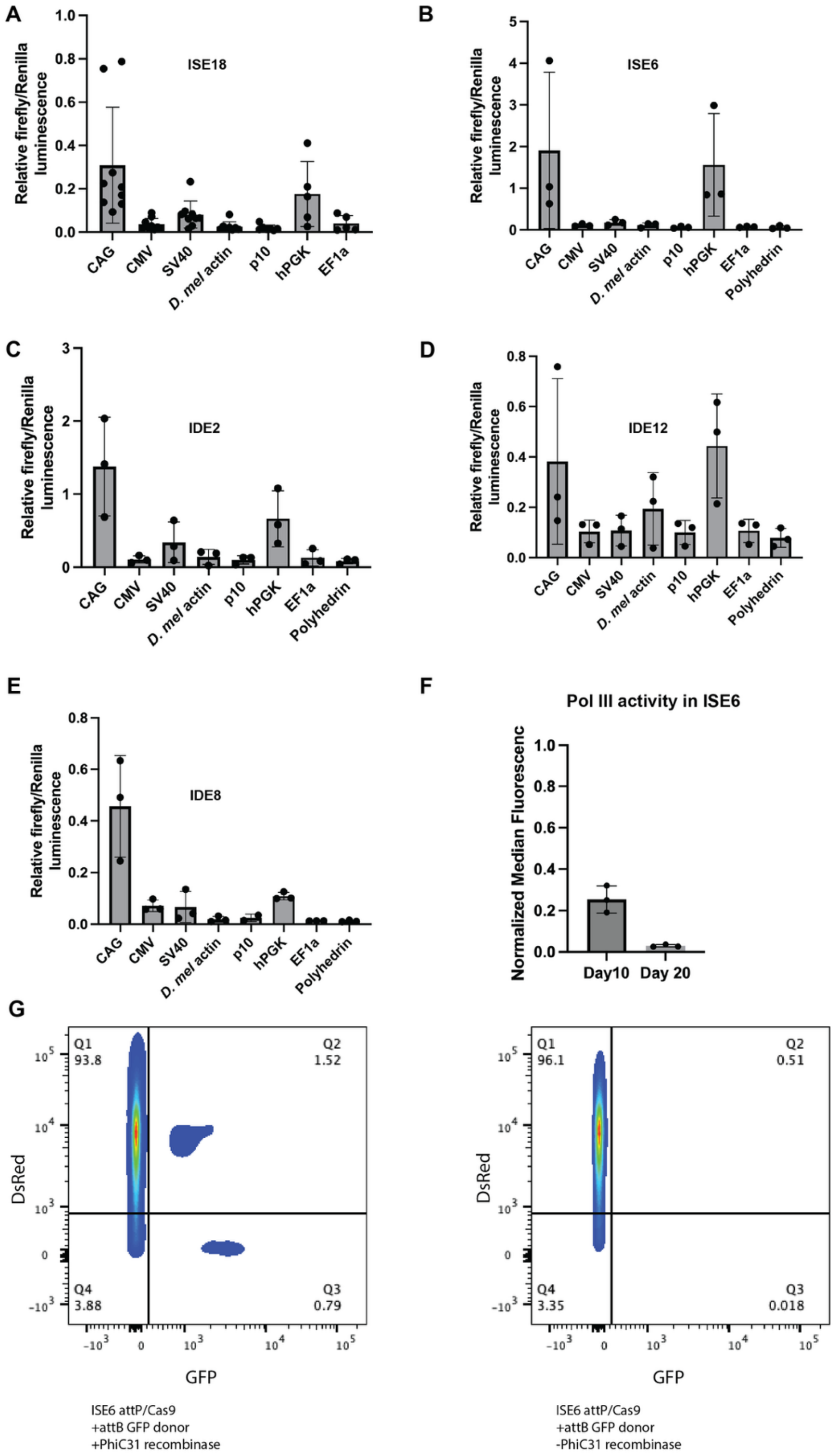

bioRxiv preprint doi: https://doi.org/10.64898/2026.05.05.721418; this version posted May 7, 2026. The copyright holder for this preprint (which was not certified by peer review) is the author/funder, who has granted bioRxiv a license to display the preprint in perpetuity. It is made available under aCC-BY 4.0 International license.

Supplemental Figure 2

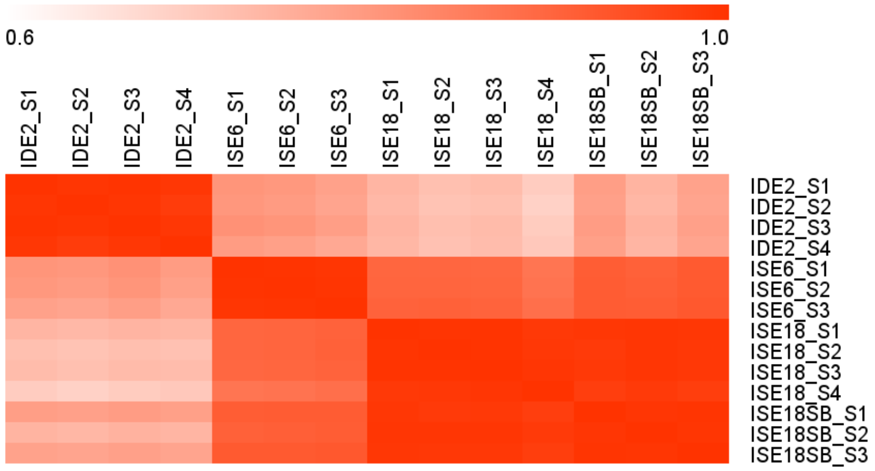

Supplemental Figure 3

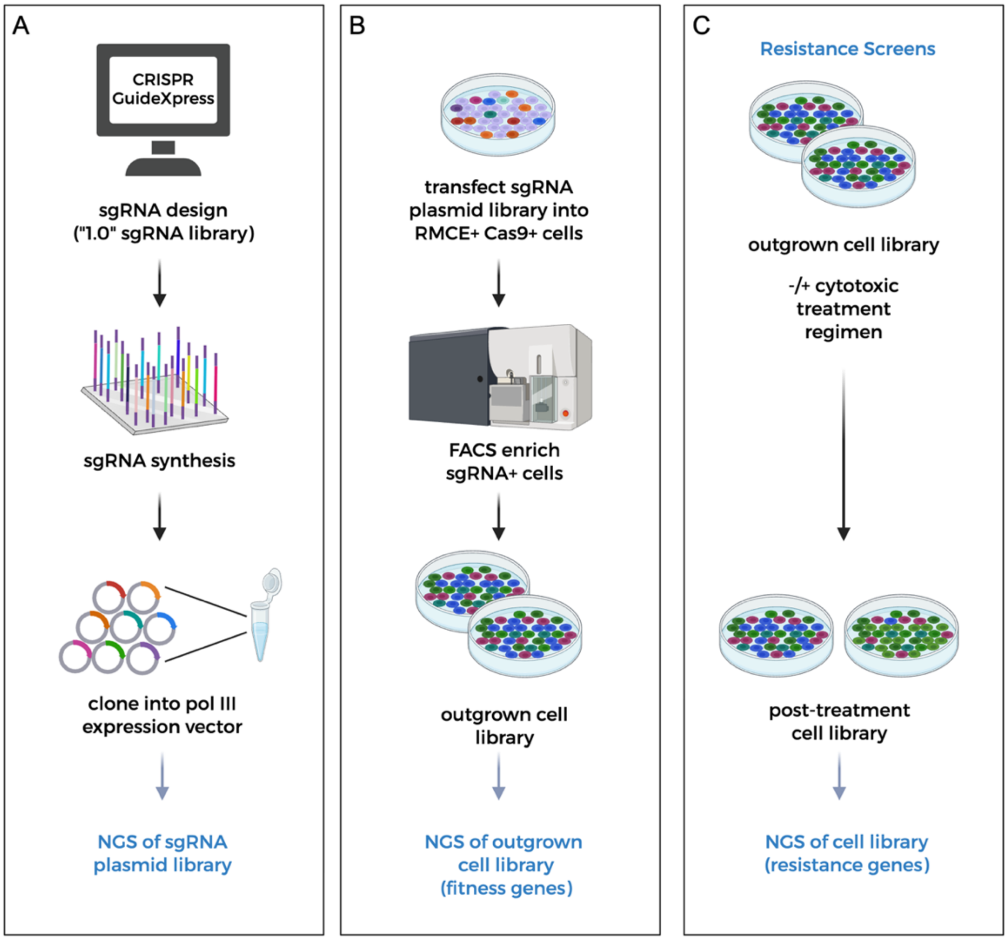

bioRxiv preprint doi: https://doi.org/10.64898/2026.05.05.721418; this version posted May 7, 2026. The copyright holder for this preprint (which was not certified by peer review) is the author/funder, who has granted bioRxiv a license to display the preprint in perpetuity. It is made available under aCC-BY 4.0 International license.

Supplemental Figure 4

A

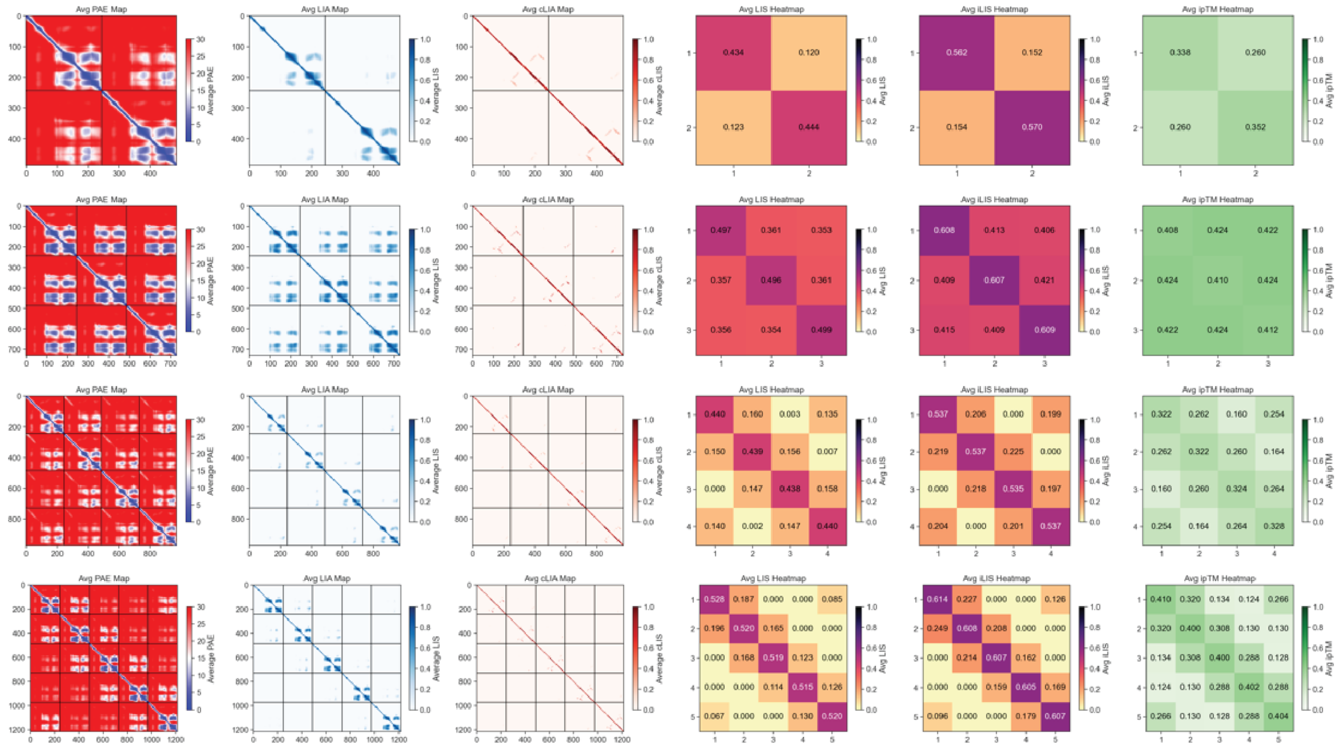

B

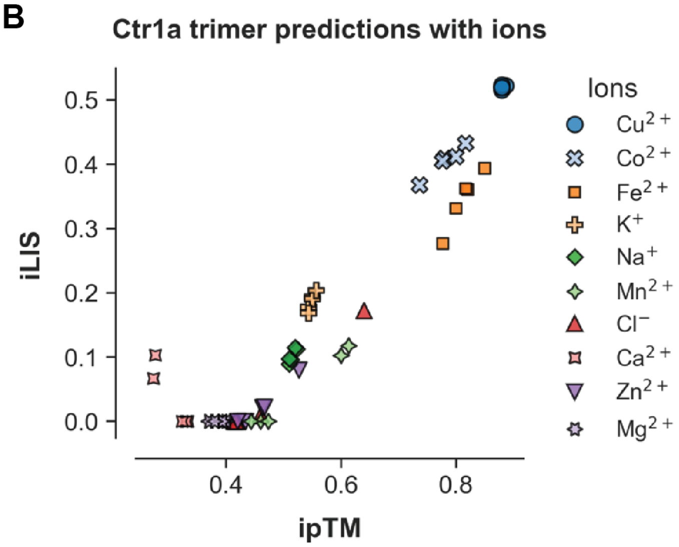

C

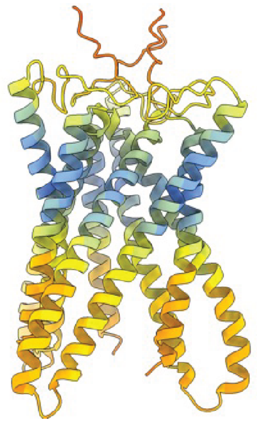

D

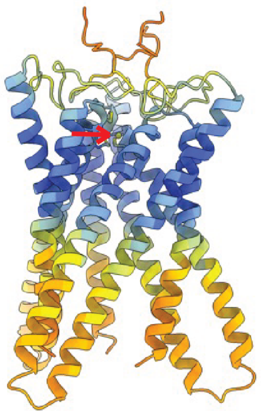

bioRxiv preprint doi: https://doi.org/10.64898/2026.05.05.721418; this version posted May 7, 2026. The copyright holder for this preprint (which was not certified by peer review) is the author/funder, who has granted bioRxiv a license to display the preprint in perpetuity. It is made available under aCC-BY 4.0 International license.

Supplemental Figure 5

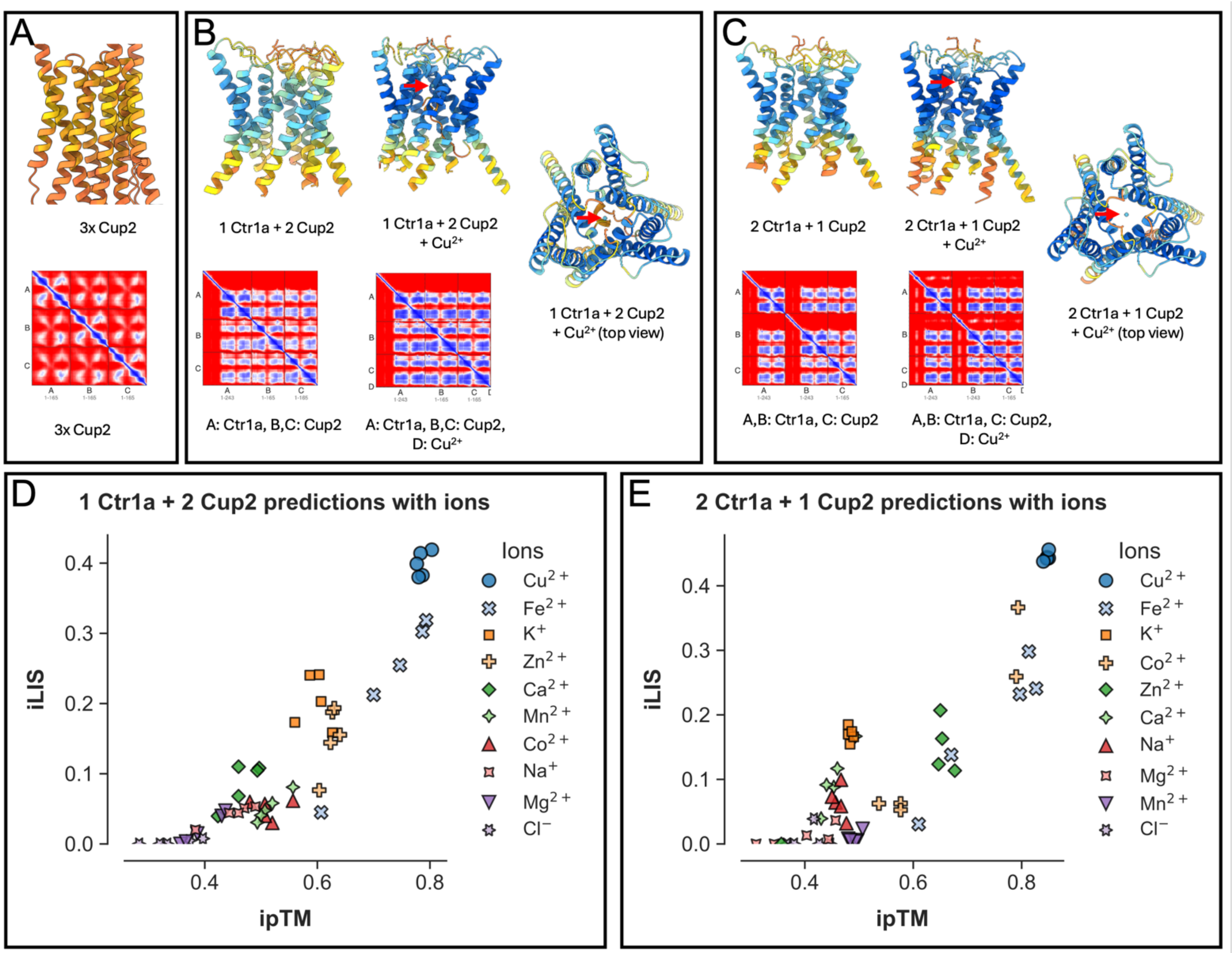

bioRxiv preprint doi: https://doi.org/10.64898/2026.05.05.721418; this version posted May 7, 2026. The copyright holder for this preprint (which was not certified by peer review) is the author/funder, who has granted bioRxiv a license to display the preprint in perpetuity. It is made available under aCC-BY 4.0 International license.

Supplemental Figure 6

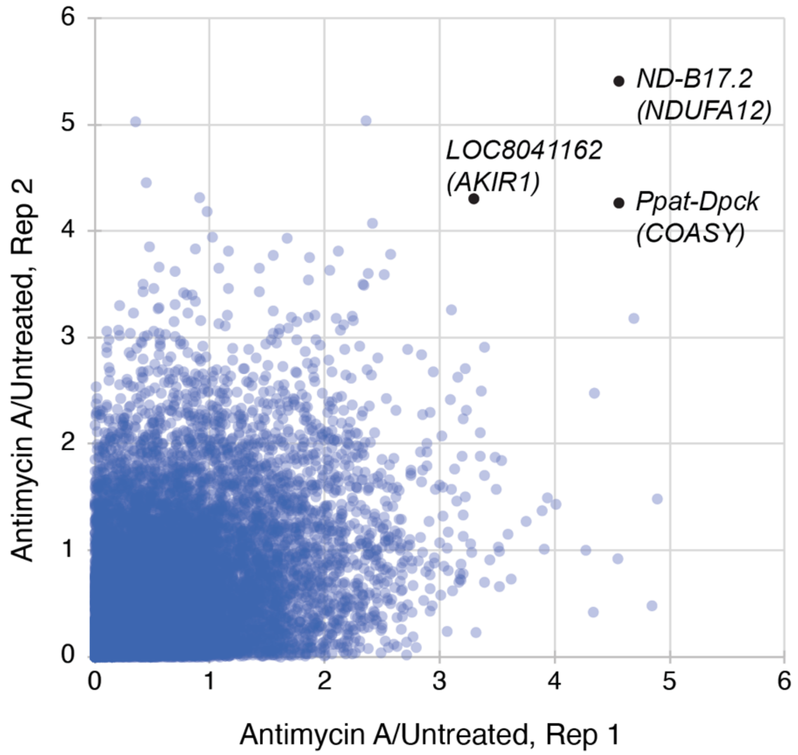

Supplemental Figure 7

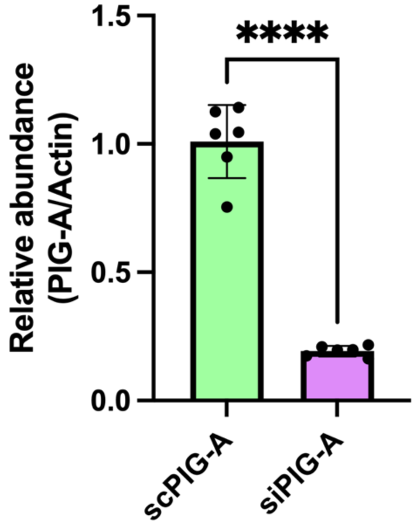

bioRxiv preprint doi: https://doi.org/10.64898/2026.05.05.721418; this version posted May 7, 2026. The copyright holder for this preprint (which was not certified by peer review) is the author/funder, who has granted bioRxiv a license to display the preprint in perpetuity. It is made available under aCC-BY 4.0 International license.

Supplemental Files

Supplemental File 1: Genome-wide *I. scapularis* CRISPR KO fitness screen dataset at sgRNA and gene levels annotated with gene expression and ortholog mapping.

Supplemental File 2: PANGEA GSEA with GO biological process and GO molecular function gene sets for the 850 *I. scapularis* fitness genes (FDR cutoff 1.0).

Supplemental File 3: PANGEA GSEAs with *Drosophila* gene sets for the *Drosophila* orthologs of the set of *I. scapularis* fitness genes (FDR cutoff 1.0).

Supplemental File 4: PANGEA GSEAs with *Drosophila* orthologs of the subset of *I. scapularis* fitness genes (FDR cutoff 0.1) that have *Drosophila* orthologs but those orthologs did not score as fitness genes in *Drosophila* S2R+ cells.

Supplemental File 5: Components of the TGF-beta/BMP signaling pathway are fitness genes in *I. scapularis* ISE18.

Supplemental File 6: PANGEA GSEAs with *I. scapularis* genes or their fly orthologs for growth-restrictive genes.

Supplemental File 7: Genome-wide *I. scapularis* CRISPR KO $CuCl_2$ resistance screen dataset.

Supplemental File 8: PANGEA GSEAs with *I. scapularis* genes for which KO confers resistance to treatment with $CuCl_2$.

Supplemental File 9: Genome-wide *I. scapularis* CRISPR KO Antimycin A resistance screen dataset.

bioRxiv preprint doi: https://doi.org/10.64898/2026.05.05.721418; this version posted May 7, 2026. The copyright holder for this preprint (which was not certified by peer review) is the author/funder, who has granted bioRxiv a license to display the preprint in perpetuity. It is made available under aCC-BY 4.0 International license.

Supplemental File 10: Genome-wide *I. scapularis* CRISPR KO Destruxin A (DA) resistance screen dataset.

Supplemental File 11: PANGEA GSEAs with *I. scapularis* genes for which KO confers resistance to treatment with Destruxin A (DA).

Supplemental File 12: Materials (cell lines, plasmids, oligonucleotide primers).
